# Gut microbiota metabolite butyric acid alleviated *Klebsiella Pneumoniae* induced lung injury by regulating CX3CR1^+^NK via PI3K/AKT pathway

**DOI:** 10.1093/burnst/tkaf069

**Published:** 2025-10-29

**Authors:** Sucheng Mu, Meijia Chang, Yongqi Shen, Xingyue Wu, Yi Han, Hao Xiang, Yue Luo, Yao Chen, Huajun Zheng, Zhenju Song, Chaoyang Tong

**Affiliations:** Department of Emergency Medicine, Zhongshan Hospital, Fudan University, 180 Fenglin Road, Shanghai 200032, China; Department of Emergency Medicine, Zhongshan Hospital, Fudan University, 180 Fenglin Road, Shanghai 200032, China; Shanghai Institute of Infectious Disease and Biosecurity, Zhongshan Hospital, Fudan University, 38 Yixueyuan Road, Shanghai 200032, China; Department of Emergency Medicine, Zhongshan Hospital, Fudan University, 180 Fenglin Road, Shanghai 200032, China; Department of Emergency Medicine, Zhongshan Hospital, Fudan University, 180 Fenglin Road, Shanghai 200032, China; Department of Emergency Medicine, Zhongshan Hospital, Fudan University, 180 Fenglin Road, Shanghai 200032, China; Department of Emergency Medicine, Zhongshan Hospital, Fudan University, 180 Fenglin Road, Shanghai 200032, China; Department of Emergency Medicine, Zhongshan Hospital, Fudan University, 180 Fenglin Road, Shanghai 200032, China; Department of Emergency Medicine, Zhongshan Hospital, Fudan University, 180 Fenglin Road, Shanghai 200032, China; Shanghai Ministry of Science and Technology Key Laboratory of Health and Disease Genomics, National Health Commission Key Laboratory of Reproduction Regulation, Shanghai Institute for Biomedical and Pharmaceutical Technologies, 2140 Xietu Road, Shanghai 200237, China; Department of Emergency Medicine, Zhongshan Hospital, Fudan University, 180 Fenglin Road, Shanghai 200032, China; Shanghai Institute of Infectious Disease and Biosecurity, Zhongshan Hospital, Fudan University, 38 Yixueyuan Road, Shanghai 200032, China; Shanghai Key Laboratory of Lung Inflammation and Injury, 180 Fenglin Road, Shanghai 200032, China; Institute of Emergency Rescue and Critical Care, Fudan University, 138 Yixueyuan Road, Shanghai 200032, China; Department of Emergency Medicine, Zhongshan Hospital, Fudan University, 180 Fenglin Road, Shanghai 200032, China

**Keywords:** *Klebsiella pneumoniae*, Acute lung injury, Gut microbiota, Butyric acid, Natural killer cells

## Abstract

**Background:**

The expression of CX3CR1 is regulated by the gut microbiota and is correlated with the prognosis of sepsis in patients. However, the underlying mechanism has remained uncertain. This study aims to explore the role of gut microbiota components in regulating CX3CR1 expression and its impact on pneumonia-induced lung injury during sepsis.

**Methods:**

Mice were fed a mixture of antibiotics to establish a pseudogerm-free mouse model and then infected with *Klebsiella pneumoniae*. Fecal microbiota transplantation (FMT) was performed on microbiota-depleted mice, and 16S rRNA gene sequencing and targeted metabolomics were used to identify the key metabolites. Flow cytometry was employed to analyze the phenotypes of natural killer (NK) cells. Butyric acid was added as a supplement for rescue. Next, NK92 cells were pretreated with butyric acid to explore the potential signaling pathways involved.

**Results:**

In the animal study, we revealed that the expression of CX3CR1 on NK cells depended on the intestinal microbiota and its metabolites, which were related to the survival rates of gut microbiota-depleted mice after *K. pneumoniae* infection. FMT increased the percentage of CX3CR1^+^ NK cells in the lungs of these mice, restored the disordered microbiota and metabolites, and alleviated the lung injury induced by infection. Among the metabolites, butyric acid was identified as the key metabolite and was shown to increase the proportion of CX3CR1^+^ NK cells and interferon (IFN)-γ secretion, reduce bacterial loads, increase lung tissue damage, and increase survival rates. In vitro, butyric acid activated the PI3K/AKT pathway in NK92 cells, promoted CX3CR1 expression, and enhanced NK cell activity and migration ability.

**Conclusions:**

We concluded that butyric acid alleviated *K. pneumoniae*-induced lung injury by regulating CX3CR1^+^ NK cells via the PI3K/AKT pathway.

Highlights

- Gut microbiota-depleted mice exhibit increased mortality after *Klebsiella pneumoniae* infection, which is rescued by fecal microbiota transplantation through the restoration of CX3CR1^+^ NK cells, highlighting the essential role of the gut microbiota in sepsis defense.
- Butyric acid, a key microbial metabolite, increases the expression of CX3CR1 on NK cells through the PI3K/AKT pathway, improving bacterial clearance, IFN-γ secretion and survival in infected mice.
- Targeted butyric acid supplementation reduces lung injury and mortality in gut microbiota-depleted mice, suggesting a potential therapeutic strategy for sepsis by that involves modulation of the gut–lung NK cell axis.

## Background

Sepsis is the leading cause of death in patients in the intensive care unit (ICU), with a fatality rate of 20%–30% [1]. According to the most recent definition published in 2016, sepsis is defined as a life-threatening syndrome caused by the dysfunctional responses of the host to infection or damage [2]. The pathogenesis of sepsis is always accompanied by intestinal injury, gut microbiota dysbiosis, and intestinal mucosal barrier dysfunction, which results from bacterial and endotoxin translocation, leading to secondary infection or even multiple organ dysfunction syndrome [3, 4]. Identifying precise diagnostic and therapeutic targets based on the pathogenesis of sepsis is crucial [5]. Currently, the interaction between host immune regulation and the gut microbiota and its metabolites has attracted increasing attention in terms of the underlying nosogenesis of sepsis [6]. It has been reported that the microbiota of septic patients is characterized by decreased alpha diversity, an increase in pathogens, and disordered metabolites [6–8]. In our previous study, we verified that the alpha diversity and short-chain fatty acid content of gut microbiota were much lower in deceased septic patients than in surviving patients [9]. The increase in the abundance of *Klebsiella* in the gut, along with the SOFA score and concentrations of taurocholic and butyric acids, were the key risk factors for death in our nomogram predictive model for septic patients [9]. Furthermore, we reported that during hospitalization, the abundance of *Klebsiella* and *Enterococcus* in the stool of septic patients increased significantly one week after admission to the ICU, at which time some patients developed secondary bloodstream or lung infections [10]. On the basis of the metagenome sequencing of pathogens from stool samples and infectious sites, we confirmed that the sequence of the secondary infectious pathogens was highly homologous to that of intestinal pathogens [10], which indicated that the secondary infectious pathogens likely originated from ever-increasing amounts of *Klebsiella* or *Enterococcus*.

Secondary infection with *Klebsiella pneumoniae* (*K. pneumoniae*) in the lung and bloodstream is among the most common causes of mortality in septic patients [11]; therefore, timely intervention at the early stage of infection is needed. *K. pneumoniae* is a gram-negative bacterium with capsules but no spores or flagella [12]. Although *K. pneumoniae* hides itself in the gastrointestinal tract in normal organisms, when an organism’s immunity is weakened or the gut microbiota is disrupted, this bacterium will multiply opportunistically and lead to severe infection [13]. Natural killer (NK) cells are among the most critical immune cells for regulating proinflammatory mediators in the early stage of *K. pneumoniae* infection [14]. Innate immune cells, including NK cells, tend to activate the production of type-1 cytokines, mediating the effective clearance of bacterial pathogens from the lung [15]. NK-secreted interferon (IFN)-γ is the essential driver of *K. pneumoniae* clearance at the early stage of infection [16] and is the key mediator for promoting the expression of IFN-γ-dependent CXC chemokines, guiding NK cell migration to infection sites to exert immunological effects [17].

The *Cx3cr1* gene is located at the chromosomal locus 3p21-3pter and encodes a seven-transmembrane G-protein-coupled receptor, which serves as the sole receptor for the chemokine CX3CL1 [18]. Upon ligand binding, CX3CR1 activation triggers intracellular signaling cascades that mediate lymphocyte migration, activation, and adhesion [19]. The CX3CL1/CX3CR1 axis regulates the release of proinflammatory mediators [20], and when the *Cx3cr1* gene is defective, secretion of tumor necrosis factor (TNF), IFN-γ, interleukin (IL)-6 and iNOS decreases markedly [21]. CX3CR1 is highly expressed on the surface of lymphocytes, especially NK cells [22], and its expression has been reported to be related to the gut microbiota and its metabolites [23, 24]. In gut microbiota-depleted mice, monocytes exhibit a decreased migratory capacity toward CX3CL1 and CCL2 ligands because of decreased CX3CR1 expression [23]. In addition, the expression level of these genes in peripheral blood monocytes is closely correlated with the prognosis of sepsis in patients [25]. However, how the gut microbiota regulates the expression of CX3CR1 on NK cells during infection remains unclear.

In this study, we further investigated the close correlation between the expression of CX3CR1 on NK cells and the gut microbiota and explored the underlying mechanism. We found that when the gut microbiota was depleted, the percentage of CX3CR1^+^ NK cells in the lung greatly decreased. On the basis of 16S rRNA gene sequencing and targeted metabolomics of stool samples from fecal microbiota transplantation (FMT)-treated microbiota-depleted mice, we hypothesized that butyric acid was the key factor promoting the expression of CX3CR1. Therefore, we aimed to investigate the effects of butyric acid on CX3CR1 and NK cells and the underlying mechanism to identify a feasible approach to protect patients infected with *K. pneumoniae* and alleviate lung injury.

## Methods

### Animal models

Specific pathogen-free C57Bl/6 mice were purchased from Shanghai Jie Si Jie Laboratory Animal Co. Ltd Mice were fed a mixture of broad-spectrum antibiotics (ampicillin 1 g/L, Macklin; neomycin sulfate 1 g/L, Macklin; metronidazole 1 g/L, Macklin; and vancomycin 0.5 g/L, CSNpharm) for two weeks to establish a pseudogerm-free mouse model [26], and the model was identified by 16S rRNA gene sequencing as described in a previous study [10]. *Cx3cr1^−/−^* mice (*Cx3cr1* knock-out mice) and wild-type C57BL/6 littermates were obtained from Shanghai Model Organisms Center (Shanghai, China). Infected mice were induced by intratracheal inoculation with 6 × 10^5^ CFU of *K. pneumoniae* (NTUH-k2044, a standardized strain with high virulence) as previously described [27]. All experiments involving live animals were conducted in compliance with the ‘Guide for the Care and Use of Laboratory Animals’ and were approved by the Institutional Review Board of Zhongshan Hospital, Fudan University, China (No. 201804001Z).

### Butyric acid supplementation

After the pseudogerm-free mouse model was established, the antibiotic water was stopped, and butyric acid (Sigma–Aldrich) was added to the drinking water at a final concentration of 10 mMol/L for microbiota-depleted mice. The cage was positioned away from light, and the drinking water supplemented with butyric acid was changed every three days for seven consecutive days.

### Flow cytometry and sorting

Single cells from tissues were resuspended and stained with fluorochrome-conjugated antibodies against CD3, CD19, NK1.1, CX3CR1, and IFN-γ (eBioscience, San Diego, CA, USA). After appropriate incubation, fixation, and washing, the samples were detected by flow cytometry (CytoFLEX S, Beckman Coulter, Inc., Brea, CA, USA or LSRFortessa X-20, BD Biosciences, San Jose, CA, USA).

For the isolation of CX3CR1^+^ NK cells from the spleen, the target resuspended cells were marked as CD3^−^NK1.1^+^CX3CR1^+^ and were sorted on a BD FACSAria II (BD Biosciences). The sorted cells were collected in sterile medium and transferred into the targeted mice within 4 h.

### Adoptive transfer of natural killer cells

The sorted CD3^−^NK1.1^+^CX3CR1^+^ NK cells were centrifuged at room temperature at 1500 rpm for 5 min and then resuspended in PBS. The cell density was adjusted to 2 × 10^6^ cells/ml, and 100 μl of the cell suspension was injected through the retrobulbous vein of mice anesthetized with Avertin.

### Deoxyribonucleic acid extraction, polymerase chain reaction amplification, and 16S ribosomal ribonucleic acid gene amplicon sequencing

Paired-end sequencing (2 × 300 bp) was performed on pooled amplicons using the Illumina MiSeq platform.

After extraction from lung, blood, and fecal stool tissues using a QIAamp DNA Stool Mini Kit (Qiagen, Hilden, Germany), the genomic DNA was detected by 1.5% agarose gel electrophoresis at room temperature. The primers with barcodes for the 16S rRNA gene amplicons were designed to target the V3-V4 hypervariable region. After amplification, the samples were purified with a QIAquick PCR Purification Kit (Qiagen, Hilden, Germany), quantified with a Qubit instrument (Life Technologies, New York, US), and then pooled at equal concentrations. The Illumina MiSeq platform was used to perform paired-end sequencing (2 × 300 bp) on pooled amplicons.

### Targeted metabolomics and data preprocessing

Targeted metabolomics technology provides broad metabolome coverage across major functional microbial metabolite classes, such as amino acids and amines, organic acids, carbohydrates, fatty acids, benzenoids, bile acids, nucleotides, sugars, vitamins, and cofactors, as measured by a triple quadrupole mass spectrometer with ultrahigh-performance liquid chromatography and Metabo-Profile Biotechnology (Shanghai) Co., Ltd

All standards were obtained from Sigma–Aldrich, Steraloids, Inc., and TRC Chemicals, weighed accurately, and a separate stock solution prepared with a concentration of 5.0 mg/ml in water, methanol, sodium hydroxide solution, or hydrochloric acid solution. An appropriate amount of each stock solution was mixed to form a stock calibration solution. The sample preparations were prepared as follows. Stool samples were thawed in an ice bath, and 10 mg was weighed and transferred to a new 1.5-ml test tube. A total of 25 μl of water was added, and the sample was homogenized with zirconia beads for 3 min. Next, 185 μl of ACN/methanol (8/2) was added to extract the metabolites, the samples were centrifuged at 18 000 × g for 20 min, and the supernatant was transferred to a 96-well plate. Newly prepared derivative reagent (20 μl per well) was added to the plate, which was derivatized for 30 min at 30°C, and the plate was subsequently diluted with 350 μl of 50% methanol solution, stored at −20°C, and centrifuged at 4000 × g and 4°C for 30 min. A total of 135 μl of the supernatant was transferred to a new 96-well plate with 15 μl of internal standards in each well. The derived reserve standard solution was added to the left well, and the plate was sealed for UPLC–MS/MS analysis. With respect to the sample analysis sequence, QC sample calibrators and blank samples were analyzed in the whole sample set. QuanMET software was used to process the raw data generated by UPLC–MS/MS, and peak integration, calibration, and quantification of each metabolite were performed. The relative concentration of butyric acid in the bronchoalveolar lavage fluid (BALF) was similarly detected by liquid chromatography–mass spectrometry (LC–MS).

### Lung colony-forming unit assay

Lung tissues from mice were diluted in tenfold normal saline and plated onto KPC media. After they were incubated aerobically at 37°C for 16 h, the colonies were counted.

### Intestinal barrier integrity

Mice were gavaged with 200 μl of Fluorescein Isothiocyanate–labeled Dextran (FITC-dextran) to assess the integrity of the intestinal barrier as previously described [28]. After 4 h, blood samples were collected by eyeball extirpation to detect the intensity of FITC-dextran in the blood. The samples were analyzed at an excitation wavelength of 480 nm, and the measurements were recorded at an emission wavelength of 520 nm. The standard substance of FITC-dextran was diluted with PBS to construct a standard curve, after which the intensity was calculated on the basis of the OD value of each sample.

### Fecal microbiota transplantation

Fecal pellets were obtained from untreated mice and resuspended in PBS (1 fecal pellet/1 ml of PBS). Several fecal pellets from different mice were collected and resuspended together in PBS. Each gut microbiota-depleted mouse was given a total of 200 μl of the resuspended fecal liquid by gavage over 2 consecutive days after two days of cessation of the antibiotic treatment.

### Culture of the natural killer 92 cell strain

Forty-five milliliters of 1640 basic medium was added to 5 ml of human AB serum (Gemini, 100–512) to prepare the NK cell medium. A total of 400 U of human IL-2 and 10 μl of triple antibody cytokine were added to the medium per milliliter. The mixture was gently mixed and stored at 4°C.

### Polymerase chain reaction

The RNA of the samples was extracted using a One-Step Mouse Genotyping Kit (Vazyme Biotech), reverse transcribed with HiScript® II Q RT SuperMix for qPCR (Vazyme Biotech), and amplified and detected via AceQ® qPCR SYBR Green Master Mix (Vazyme Biotech). The primers used were human CX3CR1-F (5′-TGGGGACATCGTGGTCTTTGGG-3′), human CX3CR1-R (5′-GGGCTTCTTGCTGTTGGTGAGG-3′), mouse CX3CR1-F (5′-TTCCCATCTGCTCAGGACCTC-3′), and mouse CX3CR1-R (5′-ATTTCCCACCAGACCGAACG-3′).

### Western blot

The protein was extracted from NK cells, and the concentration was determined using a BCA assay. After electrophoresis and transfer to a membrane, the proteins were incubated with 110-kDa PI3 Kinase p110α (CST, Inc), 110-kDa PI3 Kinase p110α (CST, Inc), 60-kDa phospho-Akt (CST, Inc), 60-kDa Akt (CST, Inc), 44-kDa CX3CR1 polyclonal antibody (Proteintech, Inc.) and 36-kDa anti-GAPDH (Abcam, Inc). The expression of the corresponding proteins was detected using a Western blot chemical imager (Clinx Science Instruments Co., Ltd).

### Small interfering ribonucleic acid transfection

For gene silencing, PI3K (NCBI Gene ID: 5291) siRNA (siPI3K) and control siRNA (NC) were designed and obtained from GenePharma (Shanghai, China). Transfection into NK92 cells was carried out using a GenePharma transfection kit according to the manufacturer’s protocols. Four siRNA sequences targeting PI3K were designed (the sequences are listed in supplementary file Table S1). The effective sequence of siPI3K was as follows: sense, 5′-3′GGCUGGUAUACAAUAACAATT; antisense, 5′-3′UUGUUAUUGUAUACCAGCCTT. The NK92 cells were cultured for another 24 h before being subjected to qPCR and for 48 h for western blot analysis.

### Detection of natural killer cell migration

All the pipette tips were precooled. The matrix was mixed with 1640 medium at a 1:10 ratio and spread on the bottom of a Transwell plate (Corning, 8-μm pore size, REF 3422). After solidification overnight, the NK cells were added to the upper wells, and the density was adjusted to 1 × 10^6^/ml. RPMI 1640 complete medium containing IL-2 (400 U/ml) supplemented with 10% human AB serum was added to the lower chamber of the Transwell plate. The cells were cultured at 37°C and 5% CO2 for 16 h, after which the cell densities from the upper and lower chambers were detected via a CCK-8 assay and are presented as the OD value. Migration rate = OD value of the lower chamber/(OD value of the upper chamber + OD value of the lower chamber) × 100%.

### Detection of natural killer cell cytotoxicity

YAC-1 cells, a lymphoma cell line widely employed as a target cell model for evaluating NK cell activity, were cocultured with NK92 cells, and the apoptosis rate was considered to indicate the cytotoxicity of the NK cells [29].

### Bioinformatics and statistical analyses

Categorical variables are presented as the N (%), and Fisher’s exact test or the χ^2^ test was used for comparison analyses. Continuous variables are presented as the mean ± SD and were compared using the Student’s t test. One-way analysis of variance was used to compare multiple groups, followed by Tukey’s post-hoc test. Data were analyzed using SPSS version 22.0 (IBM), and statistical charts were generated using Prism 7.0 (GraphPad 7.0). The paired 16S rRNA gene sequences were assembled using Mothur (version 1.41.1), and taxonomy [Operational Taxonomic Unit (OTUs), >97%] was assigned to the OTUs by comparison to the SILVA reference database (V132). After data normalization, community richness and diversity analyses (OTU; ACE, Abundance-based Coverage Estimator; Chaos index, and Shannon diversity) were performed using Mothur [30]. LEfSe was used to identify biomarkers in different groups using the linear discriminant analysis (LDA) effect value method [31]. The statistical analysis software package R (Version 3.3.1) was used for metabolite data analysis. A *P* < 0.05 was considered to indicate statistical significance.

## Results

### CX3CR1^+^ natural killer cells played a key role in mice infected with *Klebsiella pneumoniae* and decreased in gut microbiota-depleted mice

To explore the necessary role of CX3CR1 during *K. pneumoniae* infection, we generated a mouse model with global target depletion of *Cx3cr1* (Figure S1a, see online supplementary file). *Cx3cr1^−/−^* mice had a normal life expectancy; however, when infected, the mortality rate of *Cx3cr1^−/−^* mice was 100% within 72 h, while 70% of infected WT mice survived (Figure S1b, see online supplementary file). Moreover, compared with infected WT mice, *K. pneumoniae*-infected *Cx3cr1^−/−^* mice had significantly greater bacterial loads and more severe lung injury, but lower serum IFN-γ levels (Figure S1c-e, see online supplementary file). On the basis of these data, it could be hypothesized that the *Cx3cr1* gene played an important role during *K. pneumoniae* infection.

**Figure 1:**
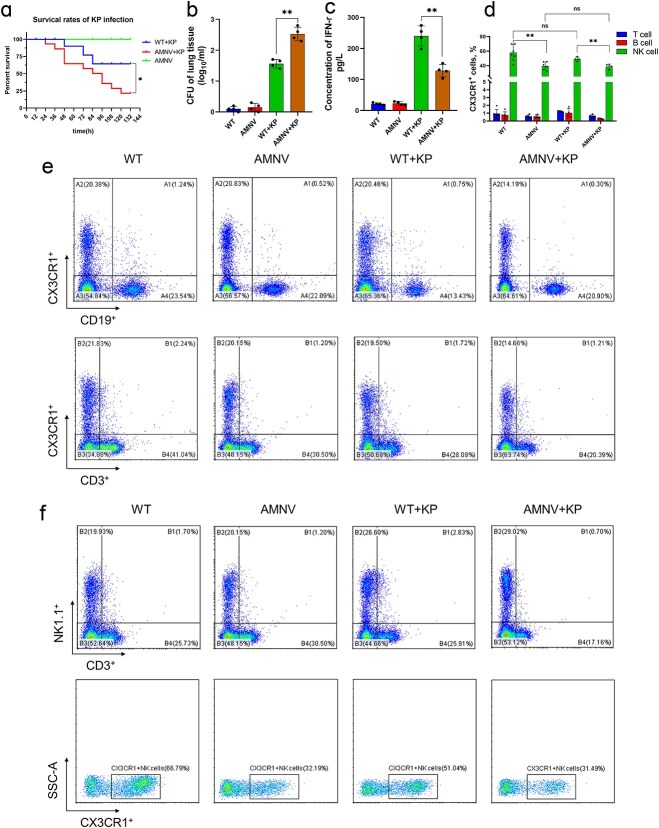
CX3CR1^+^ NK cells were reduced in microbiota-depleted mice. (**a**) Survival rate of microbiota-depleted mice infected with *K. pneumoniae* (*n* = 10–20 per group). CFU of bacterial loads in the lung (**b**), serum levels of IFN-γ (**c**), and the expression of CX3CR1 on T/B/NK cells (**d–f**) of lung tissues in microbiota-depleted mice with or without infection. ^*^P < 0.05 and ^**^P < 0.01. AMNV, mice pre-treated by ampicillin, metronidazole, neomycin, and vancomycin, indicates the microbiota-depleted mice. WT + KP, mice infected with *K. pneumoniae.* NK natural killer, *CFU* colony-forming unit, *K. pneumoniae Klebsiella pneumoniae,* AMNV *ampicillin, metronidazole, neomycin, and vancomycin pretreaed mice*, *IFN-γ* interferon-γ, *AMNV+KP* microbiota-depleted mice infected with *K. pneumoniae*

To elucidate the role of the gut microbiota during *K. pneumoniae* infection, we generated gut microbiota-depleted mice by treating them with broad-spectrum antibiotics (ampicillin, neomycin sulfate, metronidazole and vancomycin) via their drinking water. Two days after cessation of antibiotic treatment, both untreated mice (WT) and antibiotic-treated mice (AMNV) were infected with *K. pneumoniae* by intratracheal inoculation. As shown in Figure 1a, microbiota-depleted mice had a 100% survival rate, but when they were infected with *K. pneumoniae*, the mortality rate increased. In support of these findings, these microbiota-depleted mice exhibited increased bacterial loading (Figure 1b) and pathological injury in their lungs 24 h after *K. pneumoniae* infection. However, compared with that in untreated mice, the serum IFN-γ level in microbiota-depleted mice was significantly reduced (Figure 1c). We further assessed the immune cells and their CX3CR1 expression in lung tissues, and we discovered that the expression of CX3CR1 on T and B cells was very low (Figure 1d and e). CX3CR1^+^ NK cells (CD3^−^NK1.1^+^CX3CR1^+^) declined sharply in microbiota-depleted mice compared with untreated mice, either with or without *K. pneumoniae* infection (Figure 1e and f). These findings indicated that antibiotic treatment rather than *K. pneumoniae* infection caused a decrease in the proportion of CX3CR1^+^ NK cells.

### CX3CR1^+^ natural killer cells improved lung injury and increased the survival rate in gut microbiota-depleted mice infected with *Klebsiella pneumoniae*

**Figure 2:**
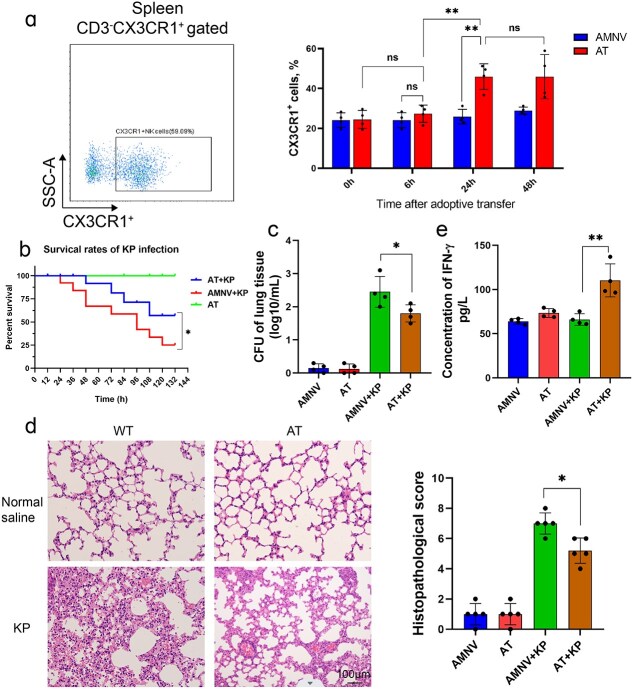
CX3CR1 played an irreplaceable role in *K. pneumoniae* infection. (**a**) After isolation of NK cells from the spleens of WT mice and adoptive transfer to microbiota-depleted mice, the percentage of CX3CR1^+^ NK cells significantly increased in AT mice, whereas the frequency in AMNV mice remained unchanged. The survival rate (**b**), CFU of bacterial loads in the lung (**c**), lung injury (**d**), and serum levels of IFN-γ (**e**) in adoptively transferred microbiota-depleted mice with or without infection. ^*^P < 0.05 and ^**^  *P* < 0.01, ns, not significant. Magnification, ×40; scale bar: 100 μm. *AT* adoptive transferred mice, *AT+KP* adoptive transferred mice infected with *K. pneumoniae, CFU* colony-forming unit, *K. pneumoniae* Klebsiella pneumoniae, *AMNV* ampicillin, metronidazole, neomycin, and vancomycin pretreaed mice, *IFN-γ* interferon-γ

We subsequently performed adoptive transfer experiments by isolating CX3CR1^+^ NK cells from the spleens of WT mice and transferring these cells into gut microbiota-depleted mice. After adoptive transfer, the percentage of CX3CR1^+^ NK cells in the gut microbiota-depleted mice markedly increased from 24.1% ± 3.7% to 45.9% ± 6.5% and was maintained at that level for at least another 24 h (Figure 2a). Therefore, *K. pneumoniae* infection of the adoptively transferred mice (AT) was performed 24 h after the adoptive transfer experiments. The mortality rate of the adoptively transferred mice decreased from 80% to 40% within 7 days after infection (Figure 2b). Compared with the untreated AMNV mice, the CX3CR1^+^ NK cell-treated mice had significantly lower bacterial loads (Figure 2c) and less injury (Figure 2d) to the lung tissue after infection. In addition, serum IFN-γ levels tended to increase (Figure 2e). Taken together, these data indicated that CX3CR1^+^ NK cells were indispensable for protecting mice from *K. pneumoniae* infection.

### Fecal microbiota transplantation eliminated the dominant *Klebsiella* in the gut and increased the number of CX3CR1^+^ NK cells in the lung

**Figure 3:**
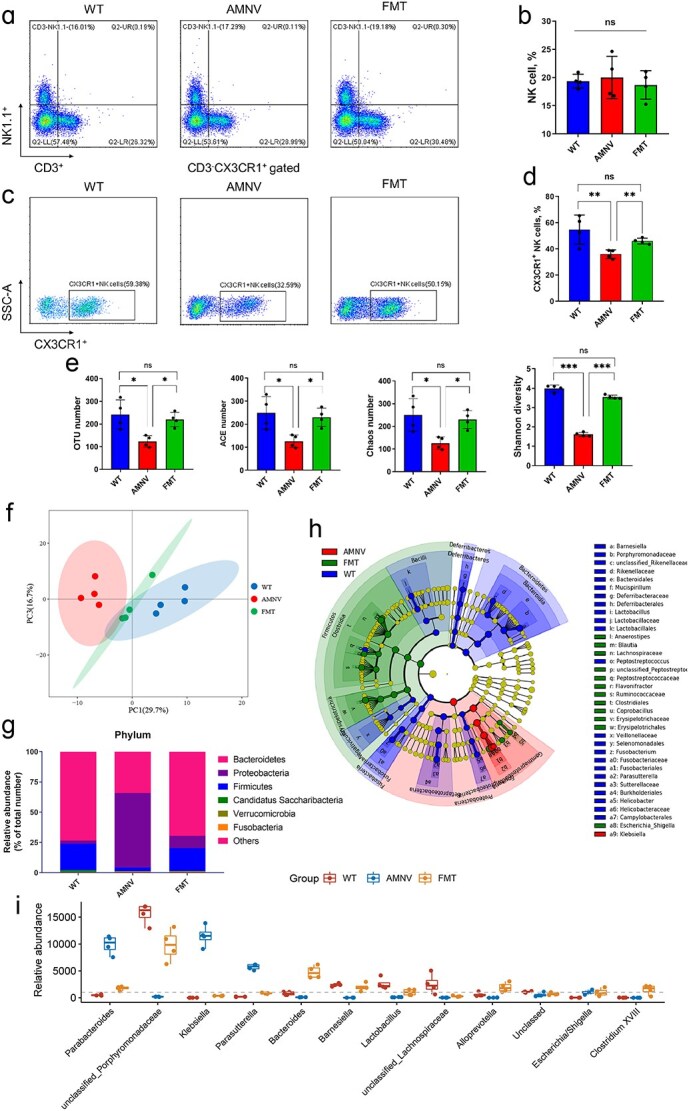
FMT restored the proportion of CX3CR1^+^ NK cells and the microbiota composition of broad-spectrum antibiotic-treated mice. The number (**a**–**d**) and proportion of CX3CR1^+^ NK cells in lung tissues from FMT-treated microbiota-depleted mice were quantified via flow cytometry. OTU, ACE, Chao, and Shannon diversity (**e**), P PCoA (**f**), mean proportions of the phylum composition (**g**), LefSE analysis (**h**), and genus composition (**i**) of the microbiota in FMT-treated microbiota-depleted mice. ‘^*^’ represents a significant difference compared with the WT group. ^*^P < 0.05, ^**^P < 0.01, and ^***^P < 0.001. ‘^#^’ represents a significant difference compared with the AMNV group. ^#^*P* < 0.05, ^##^*P* < 0.01, and ^###^*P* < 0.001, ns, not significant. *FMT* microbiota-depleted mice treated with fecal microbiota transplantation, *NK* natural killer, *OTU* operational taxonomic unit, *PCoA* principal component analysis, *ACE* Abundance-based Coverage Estimator

In light of the requirement for CX3CR1^+^ NK cells in the lung tissue of infected mice, determining the appropriate scheme or the key factor promoting the expression of CX3CR1 was considered particularly important. We then performed FMT from healthy mice to microbiota-depleted mice. Three days after FMT, we detected the percentage of NK cells in the lung tissue and the microbiota in fecal stool samples from the mice. As illustrated, the percentage of NK cells among lymphocytes showed no significant changes **(**Figure 3a and b), but the percentage of CX3CR1^+^ NK cells decreased markedly from 54.72% ± 11.12% in WT mice to 35.9% ± 3.3% in gut microbiota-depleted mice (Figure 3c and d). After FMT, the percentage of CX3CR1^+^ NK cells in microbiota-depleted mice increased to 45.9% ± 2.2%, which was significantly greater than that in non-FMT microbiota-depleted mice (Figure 3c and d). These findings suggested that the content of some components in the fecal stool of untreated mice could promote the expression of CX3CR1.

The microbiota of the fecal stools was further analyzed by 16S rRNA gene sequencing, and the OTU, ACE, Chaos, and Shannon diversity significantly decreased in microbiota-depleted mice (Figure 3e). OTU number provided direct counts of taxonomic units observed in each sample. The ACE and Chaos numbers were applied to estimate total species richness. The Shannon diversity was used to assess overall diversity by incorporating both species richness and evenness [32]. FMT reversed this trend by increasing the number of OTUs (*P* < 0.05), ACE (*P* < 0.05), Chaos (*P* < 0.05), and Shannon diversity (*P* < 0.001). A PCoA of all the samples revealed an obvious partitioning between the three groups of 46.4% (*P*_AMOVA_ < 0.01; Figure 3f). At the phylum level, the percentage of Proteobacteria prominently increased but was suppressed by FMT in microbiota-depleted mice (Figure 3g). At the genus level, the results of the LEfSe analysis revealed that the greatest potential microbial biomarker, *Klebsiella*, in microbiota-depleted mice was eliminated after FMT (Figure 3h). In addition, the relative abundances of *Parabacteroides* and *Parasutterella* decreased to normal levels, but the relative abundances of *Bacteroides* and *Lactobacillus* increased significantly (Figure 3i).

### Butyric acid increased CX3CR1^+^ natural killer cells and promoted IFN-γ secretion to combat *Klebsiella pneumoniae* infection

**Figure 4:**
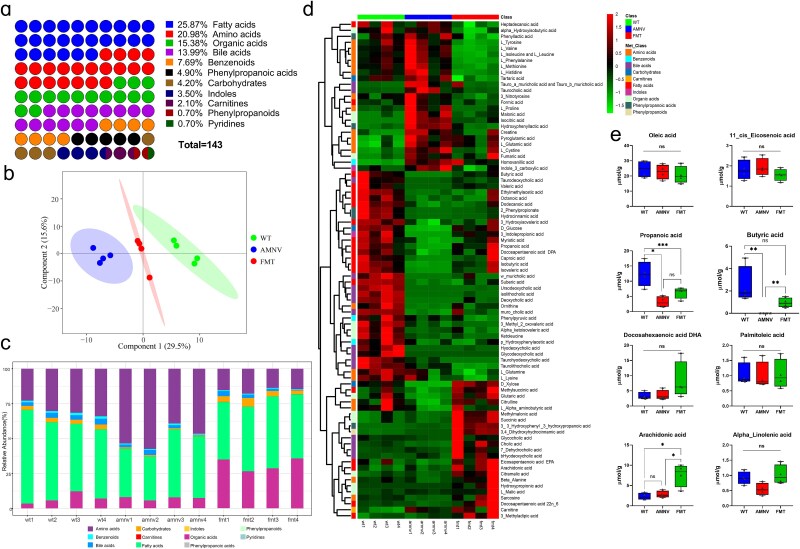
Butyric acid was the key metabolite in the FMT group. Targeted metabolomics analysis of the 143 total metabolites in the FMT samples (**a**), principal component analysis (**b**), mean proportion of 11 major classes of metabolites (**c**), heatmap of all the metabolites (**d**), and eight short-chain fatty acids (**e**). ^*^P < 0.05, ^**^P < 0.01, and ^***^P < 0.001, ns, not significant. *FMT* fecal microbiota transplantation

We further analyzed the microbiota metabolites among these mice using targeted metabolomics. A total of 143 metabolites were detected, including 37 fatty acids, accounting for 25.87% of the total (Figure 4a, Figures S2 and S3). The possible roles and biological mechanisms of the differentially abundant metabolites among the three groups were analyzed and added to Supplementary file 1 (Figure S2, see online supplementary file). The PCoA of all the samples revealed an obvious partitioning between the three groups by 45.1% (*P*_AMOVA_ < 0.001; Figure 4b). Among the 11 major classes of metabolites, fatty acids decreased most significantly in the feces of microbiota-depleted mice, and the content recovered after FMT (Figure 4c). The heatmaps (Figure 4d) and the raw data indicated that the top 8 fatty acid metabolites were oleic acid, propanoic acid, docosahexaenoic acid (DHA), arachidonic acid, 11_cis_eicosenoic acid, butyric acid, palmitoleic acid, and alpha_linolenic acid. Among the 8 top metabolites, butyric acid showed the most drastic variation, as it decreased approximately 900-fold in microbiota-depleted mice and increased 300-fold after FMT (Figure 4e). In our previous research, butyric acid was the most decreased metabolite in septic patients during hospitalization, especially in deceased septic patients [9]. Hence, we hypothesized that butyric acid was likely the key factor in the increased expression of CX3CR1.

**Figure 5:**
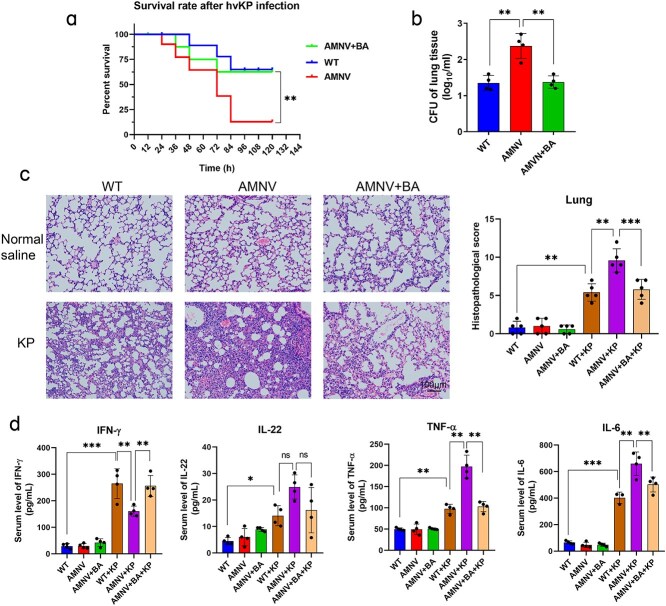
Butyric acid had a protective effect on gut microbiota-depleted mice during infection. Survival rate (**a**, *n* = 10–20 per group), CFU of bacterial loads in the lung (**b**), lung injury (**c**), and serum levels of IFN-γ, IL-22, TNF-α, and IL-6 (**d**) in butyric acid-pretreated microbiota-depleted mice infected with *K. pneumoniae*. ^*^P < 0.05, ^**^P < 0.01, and ^***^P < 0.001, ns, not significant. Magnification, ×40; scale bar: 100 μm. AMNV+BA, microbiota-depleted mice treated with butyric acid. AMNV+BA+KP, microbiota-depleted mice treated with butyric acid and then infected with *K. pneumoniae. BA* butyric acid, *CFU* colony-forming unit, *K. pneumoniae Klebsiella pneumoniae, AMNV* ampicillin, metronidazole, neomycin, and vancomycin pretreaed mice, *IFN-γ* interferon-γ, *IL* interleukin, *TNF-α*, tumor necrosis factor alpha

To verify this hypothesis, we added butyric acid to the drinking water of microbiota-depleted mice for one week and then challenged the mice with *K. pneumoniae* (6 × 10^5^ CFU). We measured butyrate levels in the BALF of the mice and found that it was nearly undetectable in microbiota-depleted mice, whereas one week of butyrate supplementation restored its level in the BALF and fecal (Figure S3a and b, see online supplementary file). In response to butyric acid supplementation, the mortality rate of microbiota-depleted mice significantly decreased to 30% during infection (Figure 5a). Moreover, the bacterial loads (Figure 5b) and injury (Figure 5c) in the lung tissue of butyric acid-supplemented microbiota-depleted mice were dramatically lower than those in the lung tissue of nonbutyric acid-supplemented mice. The levels of the inflammatory factors TNF-α and IL-6 were also significantly higher in infected microbiota-depleted mice than in infected WT mice (Figure 5d), and butyric acid supplementation reduced the serum concentrations of these two inflammatory factors.

**Figure 6:**
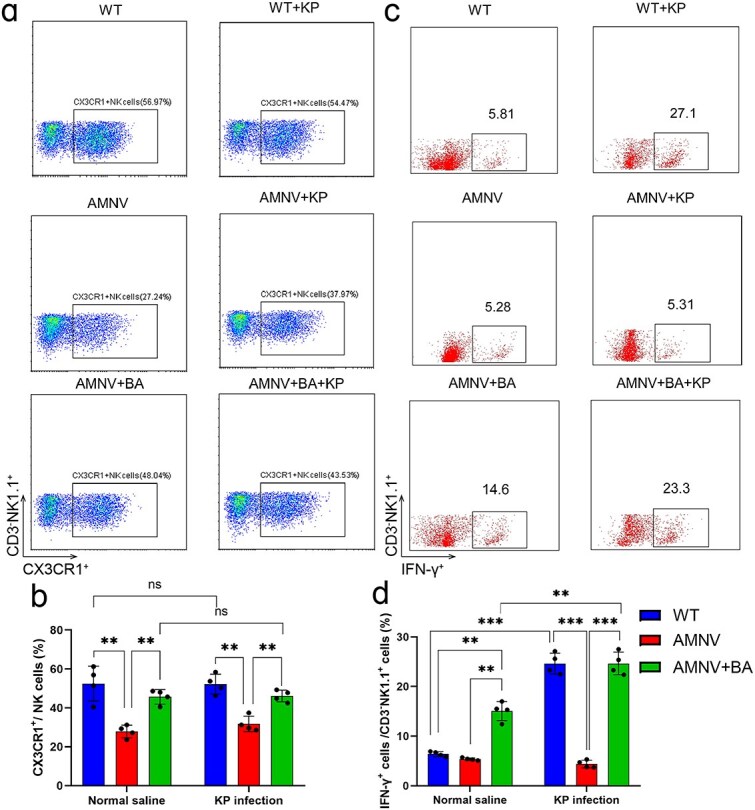
Butyric acid promoted the secretion of IFN-γ by NK cells. The proportion of CD3^−^NK1.1^+^CX3CR1^+^ NK cells (**a** and **b**) in lung tissues from butyric acid-pretreated microbiota-depleted mice. The secretion of the intracellular cytokine IFN-γ in butyric acid-pretreated microbiota-depleted mice (**c** and **d**). ^**^P < 0.01 and ^***^  *P* < 0.001, ns, not significant. *IFN-γ* interferon-γ, *NK* natural killer

As protective factors against *K. pneumoniae*, IFN**-γ** and IL-22 levels increased in WT mice during infection [14, 33]. In microbiota-depleted or *Cx3cr1^−/−^* mice, the serum IFN-**γ** level failed to increase to an adequate level similar to that in WT mice during infection (Figure 1c, Figure S1c, see online supplementary file), whereas in butyric acid-pretreated microbiota-depleted mice, the serum level of IFN**-γ** was elevated. To further elucidate the function of butyric acid, we detected intracellular cytokine levels via flow cytometry. As shown in Figure 6a and b, the percentage of CX3CR1^+^ NK cells was increased by butyric acid in both infected and noninfected mice. Furthermore, the percentage of IFN-γ^+^ NK cells significantly increased in the lung tissue of WT mice infected with *K. pneumoniae* in but not in microbiota-depleted mice (Figure 6c and d). With the addition of butyric acid to drinking water, the microbiota-depleted mice exhibited a greater percentage of IFN-γ^+^ NK cells under noninfected conditions, and the percentage was much greater during *K. pneumoniae* infection of microbiota-depleted mice that received butyric acid compared with those that did not (*P* < 0.001; Figure 6d).

### Butyric acid increased CX3CR1 expression and natural killer cell viability via the PI3K/AKT signaling pathway

**Figure 7:**
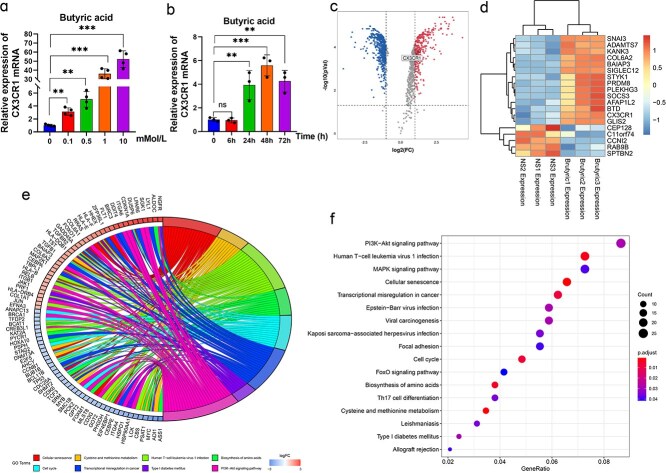
Butyric acid promoted CX3CR1 expression by activating the PI3K/AKT pathway in NK92 cells. The expression of CX3CR1 affected by different times and concentrations of butyric acid (**a** and **b**) on the expression of CX3CR1 in the medium of NK92 cells. Analysis of the mRNA sequencing results of NK92 cells pretreated with butyric acid for 24 h by volcano plots (**c**, |logFC| ≥ 1; red pots indicate upregulated genes, while blue ones signified down-regulated genes) and a heatmap (**d**), Chordal graph (**e**), and KEGG analysis (**f**) of the enrichment of the most differentially expressed genes. ^**^P < 0.01 and ^***^P < 0.001, ns, not significant. *PI3K* Phosphoinositide 3-Kinase, *KEGG* Kyoto Encyclopedia of Genes and Genomes, *AKT* protein kinase B, *NK* natural killer

All the findings presented above indicated that the gut microbiota metabolite butyric acid could increase the percentage of CX3CR1^+^ NK cells in the lung tissue of mice and increase the secretion of IFN-γ by NK cells. In vitro, we further revealed that the expression of CX3CR1 on NK92 cells was butyric acid time- and concentration dependent but showed no association with the antibiotics that were used to build the model of microbiota-depleted mice (Figure 7a and b, Figure S4a-d, see online supplementary file). The acidic properties of butyric acid affected the proliferation of NK-92 cells. Therefore, we selected the lowest butyric acid concentration (0.1 mMol/L) that affected CX3CR1 expression as the final concentration. To elucidate the underlying mechanism by which butyric acid promoted the expression of CX3CR1 on NK cells, we performed mRNA sequencing of NK92 cells pretreated with butyric acid for 24 h. As shown in Figure 7c and d, 849 genes were downregulated in butyric acid-pretreated NK92 cells and 2191 genes were upregulated, including the *Cx3cr1* gene. Kyoto Encyclopedia of Genes and Genomes (KEGG, Figure 7e) analysis revealed that the PI3K/AKT signaling pathway was enriched with the most differentially expressed genes, the majority of which were upregulated (Figure 7f).

**Figure 8:**
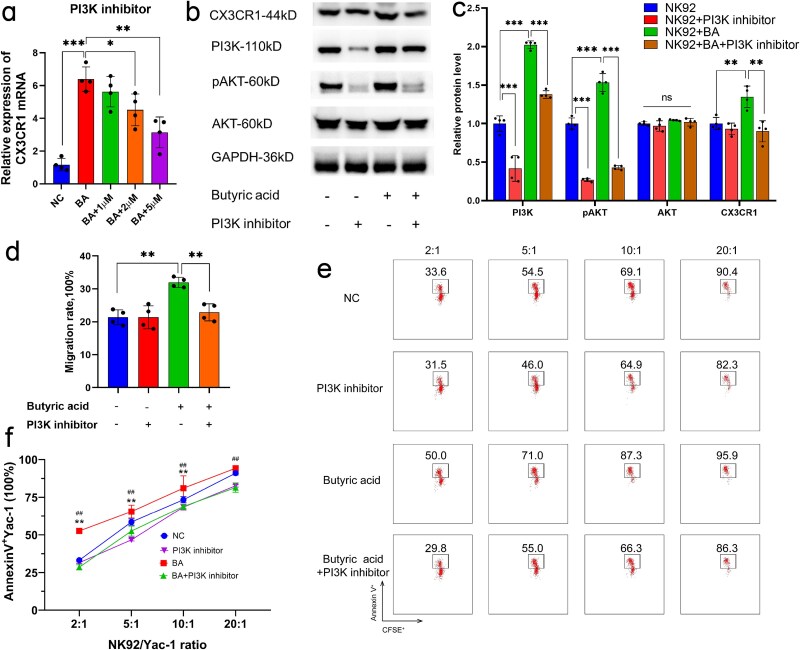
Butyric acid enhanced NK cell activity and improved the NK cell killing and migration abilities. The mRNA (**a**) and protein (**b** and **c**) expression of CX3CR1 in NK92 cells pretreated with a PI3K inhibitor. The migration rate of NK92 cells (**d**) and the rate of YAC-1 cell apoptosis rate induced by NK92 cells (**e** and **f**) pretreated with a PI3K inhibitor. NC represents negative control. ^*^P < 0.05, ^**^P < 0.01, and ^***^P < 0.001, ns, not significant. *NK* natural killer, *PI3K* Phosphoinositide 3-Kinase

The mRNA sequencing results revealed that the effect of butyric acid on the expression of CX3CR1 was likely associated with the PI3K/AKT signaling pathway. Thus, we used a PI3K inhibitor to block this signaling pathway. The expression of CX3CR1 was affected by the PI3K inhibitor in a concentration-dependent manner (Figure 8a), and we chose a final concentration of 5 μM for further verification. Butyric acid significantly upregulated phospho-AKT (p-AKT), PI3K, and CX3CR1 protein expression, as evidenced by Western blot analysis (Figure 8b and c). Critically, these increases were abolished upon cotreatment with a PI3K inhibitor. To further validate the central role of PI3K, we knocked down PI3K in NK92 cells via RNA interference (Figure S4a and b, see online supplementary file). Consistent with the inhibition results, butyric acid-induced upregulation of p-AKT and CX3CR1 expression was markedly attenuated in PI3K-deficient cells (Figure S5a-d, see online supplementary file).

Since the chemokine receptor CX3CR1 mediates the migration of NK cells, we detected the migration ability of NK92 cells using the Transwell assays. The migration rate of NK92 cells pretreated with butyric acid significantly increased from 21.37% ± 2.29% to 31.96% ± 1.55% (Figure 8d), which was also abolished by the PI3K inhibitor. To determine the cytotoxicity of NK cells, CFSE-marked Annexin V^+^ YAC-1 cells were detected by flow cytometry after coculture with NK cells, and the apoptosis rate of the YAC-1 cells was used to determine the cell viability and cytotoxicity of the NK cells. As presented in Figure 8e and f, as the ratio of NK92 cells to YAC-1 cells rose, the proportion of apoptosis of YAC-1 cells gradually increased. In addition, butyric acid significantly increased the percentage of CFSE^+^ Annexin V^+^ YAC-1 cells, and this increase was suppressed by a PI3K inhibitor. In vitro, we observed that PI3K inhibition markedly attenuated the protective effects of butyric acid in mice, accompanied by increased bacterial CFUs in lung tissue and significantly reduced CX3CR1 expression on NK cells. These findings aligned with the cellular-level experimental conclusions and are shown in Supplementary file 1 (Figure S6, see online supplementary file).

## Discussion

In the current study, we revealed that the proportion of CX3CR1^+^ NK cells decreased in mice with antibiotic-induced microbiota depletion, and the mortality rate increased when the animals were infected with *K. pneumoniae*. FMT was performed on microbiota-depleted mice, and butyric acid was identified as the key metabolite that restored the expression of CX3CR1 on NK cells and alleviated *K. pneumoniae*-induced lung injury*.* In vitro, we revealed that butyric acid activated the PI3K/AKT signaling pathway to improve the cell viability, cytotoxicity, and migration of NK cells to eliminate *K. pneumoniae.*

*Cx3cr1* was reported to be one of the genes most differentially expressed between survivors and non-survivors in two independent cohorts of septic patients and was proposed as a marker of sepsis-induced immunosuppression [34]. If monitored, the host’s molecular biomarker response could be beneficial for the identification of patients at high risk of death in the ICU [25]. CX3CR1 has also been reported to be predominantly expressed on terminally differentiated subset KLRG1^+^ NK cells [35] and is considered crucial for NK cell recruitment or activation from bone marrow to extramedullary tissues during the onset of inflammation [36]. In vivo, we showed that the percentage of CX3CR1^+^ NK cells in lung tissue was greatly reduced in microbiota-depleted mice and that the mortality rate increased in mice infected with *K. pneumoniae*, indicating that CX3CR1 was irreplaceable in infection-induced lung injury and that its expression was related to the gut microbiota. Moreover, adoptive transfer of CX3CR1^+^ NK cells into microbiota-depleted mice reduced mortality, further revealing that CX3CR1^+^ NK cells played an indispensable role in *K. pneumoniae*-induced lung injury. Previously, transplantation of NK cells has been applied in the treatment of antitumor immunity [37–39], antivirus effects [40–42], immune regulation and anti-infection effects [33, 43, 44]. Our study suggested the imperative effect of CX3CR1^+^ NK cells on bacterial clearance or even prognosis in the early stage of *K. pneumoniae* infection.

Research has reported that the gut microbiota participates in modulating inflammatory responses, including the function of myeloid cells in bone marrow and extramedullary organs [27, 45]. The loss of the gut microbiota and metabolites impaired the phagocytosis of pathogens by monocytes [46, 47], and dietary supplementation with *Clostridium butyricum* or butyric acid rescued the maturation and function of NK cells [48]. In our study, microbiota-depleted mice had severe lung injury and a high bacterial burden because of the low percentage of CX3CR1^+^ NK cells. FMT or butyric acid supplementation mitigated injury and inflammatory responses by clearing *K. pneumoniae* and reducing the levels of TNF-α and IL-6. A recent study revealed that butyric acid reversed the restriction on NK cell function by inhibiting mTORC1 and c-Myc mRNA expression to prevent hyperinflammatory responses [49], supporting the potential role of butyric acid.

In butyric acid-pretreated NK cells, genes related to the PI3K/AKT signaling pathway, one of the most critical pathways in the development and physiological functions of NK cells, were enriched [50, 51]. PI3K family members include three classes according to their homology and substrate specificity [52], and PI3K class I members contain several other domains that regulate the localization and function of the p85/p110 heterodimer, which is involved in the differentiation, proliferation, killing, and migration of NK cells [53]. It has been reported that the activation of PI3K promotes the release of IFN-γ [54] and the initiation of cellular activity, such as cytotoxicity, in NK cells [55]. In our study, PI3K and phospho-AKT were activated, and the expression of CX3CR1 was promoted by butyric acid in NK cells, which was blocked by a PI3K inhibitor, which indicated that *Cx3cr1* was likely the target gene. These findings were consistent with the active function of butyric acid toward the PI3K/AKT pathway identified in microglia by Genevieve Saw [56] and in neuronal cells by Zhou [57]. A previous study revealed that the cytoactivity of NK cells was suppressed by butyric acid [58, 59]; however, our findings revealed that butyric acid-pretreated NK92 cells presented increased migration, cell viability, and cytotoxicity, in line with the findings that the viability of NK cells could be suppressed when the PI3K/AKT signaling pathway was blocked by Tim-3, as reported by Siyu Tan and colleagues [51].

Previous research has suggested that IFN-γ released by NK cells affects macrophage [16] or neutrophil [43] activity. We determined that the secretion of INF-γ was strongly correlated with that of CX3CR1 in *K. pneumoniae*-infected mice. Some researchers have considered butyric acid to have an inhibitory effect on IFN-γ [60, 61]. These studies used exogenous IFN-γ to induce cell injury, after which butyric acid was introduced to intervene in the downstream signaling pathway. However, there was no evidence for the effect of butyric acid on the production and secretion of IFN-γ. In contrast, Thomas E Weber reported that porcine PBMCs isolated from venous blood and stimulated with concanavalin A showed cAMP-dependent increases in IL-10 and IFN-γ levels due to the activation of butyric acid [62]. In the current study, mice infected with *K. pneumoniae* had higher serum IFN-γ levels, but when the microbiota or its metabolites were absent, the serum IFN-γ level failed to increase despite infection. After butyric acid pretreatment, the serum IFN-γ concentration increased to the corresponding level in WT mice, indicating that butyric acid positively affected IFN-γ.

There are three limitations of our study. The microbiota-depleted mice were not germ-free mice because of the strict breeding environment; therefore, some physiological or pathological phenotypes were probably biased. Second, validation of the IFN-γ pathway for NK cells against *K. pneumoniae* is needed. Third, technical constraints prevented successful primary lung NK cell experiments, and further studies should optimize tissue-specific NK isolation protocols. Despite these limitations, our study provides additional evidence, and we believe that butyric acid, a gut microbiota metabolite, could be a promising supplement to improve immunity in patients with secondary infections in the ICU.

## Conclusions

CX3CR1^+^ NK cells were decreased in microbiota-depleted mice and were indispensable for combatting *K. pneumoniae* infection. FMT or butyric acid could restore the percentage of CX3CR1^+^ NK cells, increase the secretion of IFN-γ, alleviate lung injury, and restrict inflammatory responses in microbiota-depleted mice to increase mortality rates. In vitro, the expression of CX3CR1, cell viability, cytokine ability, and the migration rate of NK cells were enhanced by butyric acid via the PI3K/AKT signaling pathway. The expression of CX3CR1 on NK cells could be a potential biomarker and target for the treatment of septic patients with *Klebsiella* infection in the future.

## Supplementary Material

Figure_S1_tkaf069

Figure_S2_tkaf069

Figure_S3_tkaf069

Figure_S4_tkaf069

Figure_S5_tkaf069

Figure_S6_tkaf069

Table_S1_tkaf069

## Data Availability

The raw 16S rRNA gene sequencing data of the mice and the RNAseq data of the NK92 cells have been submitted to the Sequence Read Archive (SRA) under accession number PRJNA1009430. The remaining data are available from the corresponding author upon request.
