# Supplementary material for: Gut microbiota metabolite butyric acid alleviated Klebsiella Pneumoniae induced lung injury by regulating CX3CR1+NK via PI3K/AKT pathway
Source: Burns Trauma. 2025 Oct 29;14:tkaf069. doi: 10.1093/burnst/tkaf069 (PMC12794618; doi:10.1093/burnst/tkaf069)
Supplement: Figure_S1_tkaf069 [file figure_s1_tkaf069.pdf]

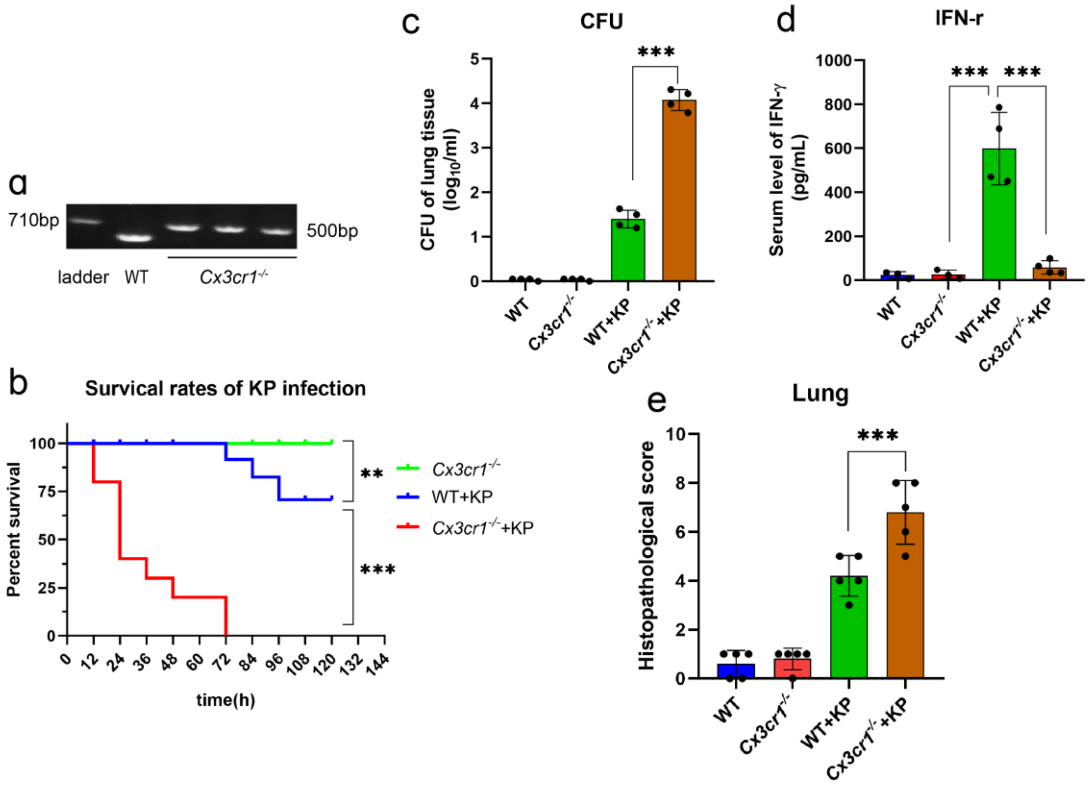

Figure S1. The protective role of CX3CR1 on mice infected with *K. pneumoniae*. DNA agarose gel electrophoresis (a), survival rate (**b**, n=10-20 per group), CFU of bacterial loads in the lung (**c**), serum levels of IFN-γ (**d**), lung injury histopathological score (**e**) of *Cx3cr1*$^{-/-}$ mice infected with *K. pneumoniae*. * indicates $P < 0.05$, ** indicates $P < 0.01$, ***, indicates $P < 0.001$
