# Supplementary material for: Gut microbiota metabolite butyric acid alleviated Klebsiella Pneumoniae induced lung injury by regulating CX3CR1+NK via PI3K/AKT pathway
Source: Burns Trauma. 2025 Oct 29;14:tkaf069. doi: 10.1093/burnst/tkaf069 (PMC12794618; doi:10.1093/burnst/tkaf069)
Supplement: Figure_S2_tkaf069 [file figure_s2_tkaf069.pdf]

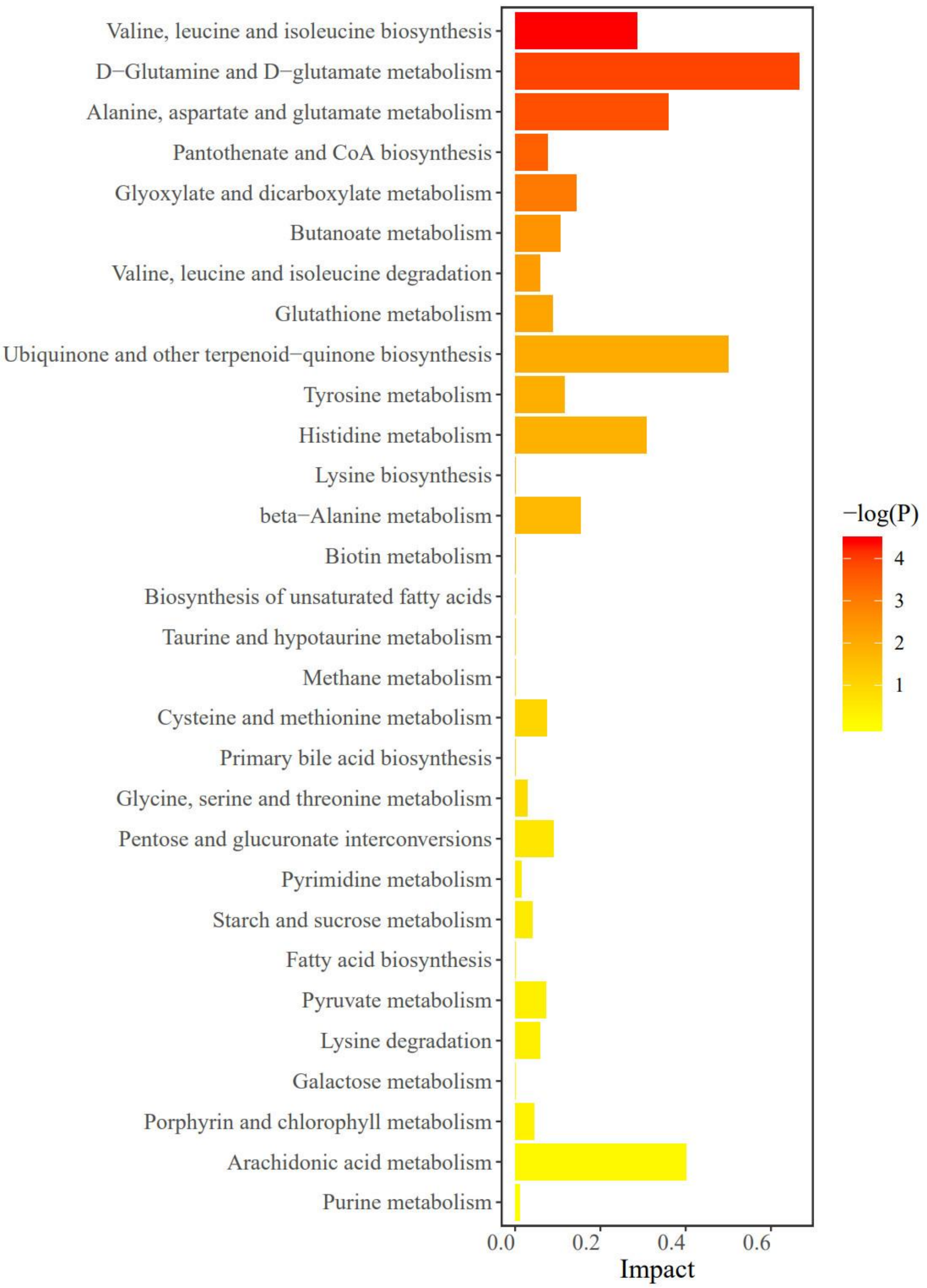

Figure S2. Pathway analysis of differential metabolites among three groups. The color of different depths represents the different *P* value, and the darker the color, the more significant the difference. The length of bar (the impact index) represented the related metabolites enriched in the pathway
