# Supplementary material for: Gut microbiota metabolite butyric acid alleviated Klebsiella Pneumoniae induced lung injury by regulating CX3CR1+NK via PI3K/AKT pathway
Source: Burns Trauma. 2025 Oct 29;14:tkaf069. doi: 10.1093/burnst/tkaf069 (PMC12794618; doi:10.1093/burnst/tkaf069)
Supplement: Figure_S3_tkaf069 [file figure_s3_tkaf069.pdf]

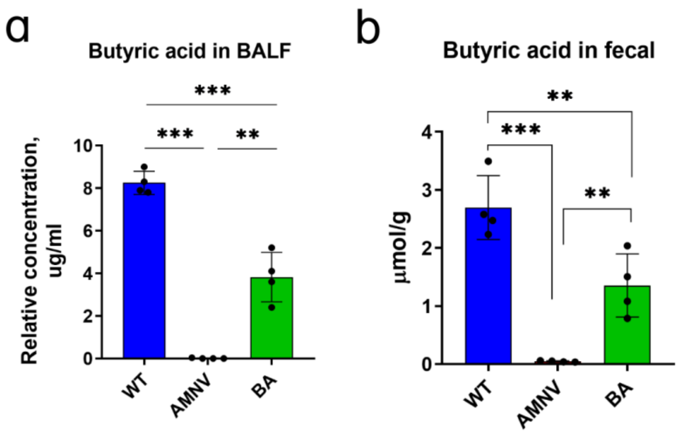


**Figure S3.** Determination of butyric acid levels in mouse bronchoalveolar lavage fluid (a) and feces (b) after oral butyrate supplementation
