# Supplementary material for: Gut microbiota metabolite butyric acid alleviated Klebsiella Pneumoniae induced lung injury by regulating CX3CR1+NK via PI3K/AKT pathway
Source: Burns Trauma. 2025 Oct 29;14:tkaf069. doi: 10.1093/burnst/tkaf069 (PMC12794618; doi:10.1093/burnst/tkaf069)
Supplement: Figure_S5_tkaf069 [file figure_s5_tkaf069.pdf]

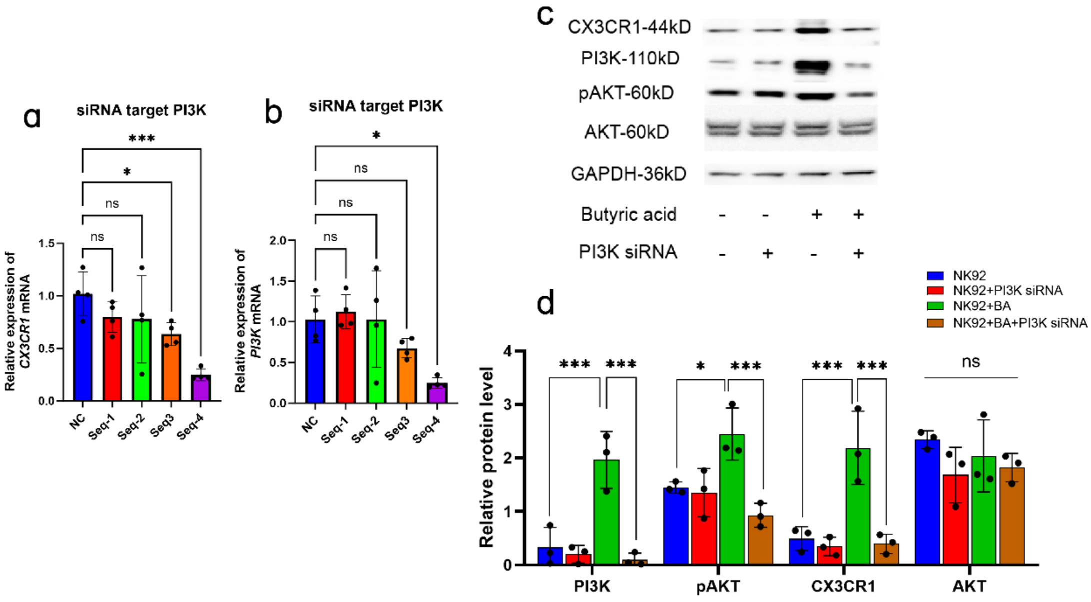

Figure S5. Expression of CX3CR1 in NK92 cells pretreated with siRNA of PI3K. Four pairs of siRNA sequences targeting PI3K (Gene ID: 5291) and validated the efficiency in NK92 cells (a&b). Subsequent experiments utilized Seq-4 for siRNA transfection. The increases in phospho-AKT and CX3CR1 by butyric acid were blocked (c&d)
