# Supplementary material for: Gut microbiota metabolite butyric acid alleviated Klebsiella Pneumoniae induced lung injury by regulating CX3CR1+NK via PI3K/AKT pathway
Source: Burns Trauma. 2025 Oct 29;14:tkaf069. doi: 10.1093/burnst/tkaf069 (PMC12794618; doi:10.1093/burnst/tkaf069)
Supplement: Figure_S6_tkaf069 [file figure_s6_tkaf069.pdf]

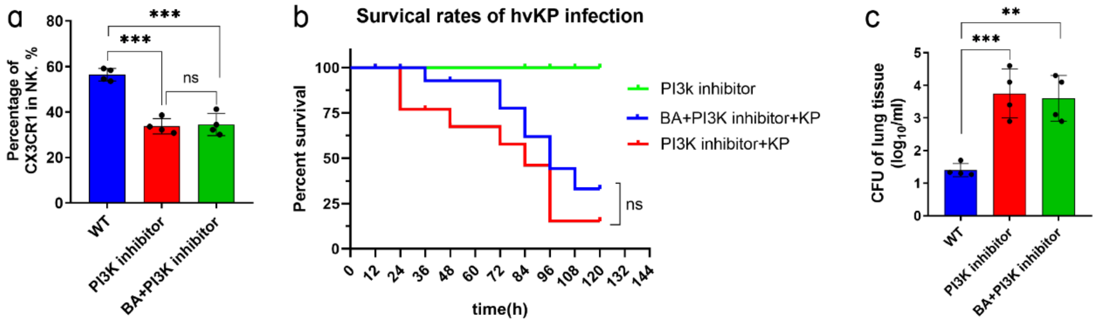

Figure S6. The expression of CX3CR1 on NK cells (a) of lung tissues**,** survival rate (b, n=10-20 per group), CFU of bacterial loads in the lung (c) among PI3K inhibitor injected mice with or without *K. pneumoniae*
