# Supplementary material for: Gut microbiota metabolite butyric acid alleviated Klebsiella Pneumoniae induced lung injury by regulating CX3CR1+NK via PI3K/AKT pathway
Source: Burns Trauma. 2025 Oct 29;14:tkaf069. doi: 10.1093/burnst/tkaf069 (PMC12794618; doi:10.1093/burnst/tkaf069)
Supplement: Table_S1_tkaf069 [file table_s1_tkaf069.pdf]

Table S1. Candidates of siRNA sequences targeting PI3K (Gene ID: 5291)

Gene		siRNA sequences
PI3K-1	sense 5'-3'	CAGCGGGAGAGUAGAAUAUTT
	antisense 5'-3'	AUAUUCUACUCUCCCGCUGTT
PI3K-2	sense 5'-3'	GGGAACUGUUCAAACAAAUTT
	antisense 5'-3'	AUUUGUUUGAACAGUUCCCTT
PI3K-3	sense 5'-3'	CCUGUAGCGUGGGUAAAUATT
	antisense 5'-3'	UAUUUACCCACGCUACAGGTT
PI3K-4	sense 5'-3'	GGCUGGUAUACAAUAACAATT
	antisense 5'-3'	UUGUUAUUGUAUACCAGCCTT
